# Hearing Screening Combined with Target Gene Panel Testing Increased Etiological Diagnostic Yield in Deaf Children

**DOI:** 10.1155/2021/6151973

**Published:** 2021-07-23

**Authors:** Le Xie, Yue Qiu, Yuan Jin, Kai Xu, Xue Bai, Xiao-Zhou Liu, Xiao-Hui Wang, Sen Chen, Yu Sun

**Affiliations:** Department of Otorhinolaryngology, Union Hospital, Tongji Medical College, Huazhong University of Science and Technology, Wuhan 430022, China

## Abstract

Genetic testing is the gold standard for exploring the etiology of congenital hearing loss. Here, we enrolled 137 Chinese patients with congenital hearing loss to describe the molecular epidemiology by using 127 gene panel testing or 159 variant testing. Sixty-three deaf children received 127 gene panel testing, while seventy-four patients received 159 variant testing. By use of 127 gene panel testing, more mutant genes and variants were identified. The most frequent mutant genes were *GJB2*, *SLC26A4*, *MYO15A*, *CDH23*, and *OTOF*. By analyzing the patients who received 127 gene panel testing, we found that 51 deaf children carried variants which were not included in 159 variant testing. Therefore, a large number of patients would be misdiagnosed if only 159 variant testing is used. This study highlights the advantage of 127 gene panel testing, and it suggests that broader genetic testing should be done to identify the genetic etiology of congenital hearing loss.

## 1. Introduction

Congenital hearing loss (HL) is a common disease, and about 1 to 2 per 1000 live births suffer from congenital hearing loss in the world. The prevalence of hearing loss increases with age. Among the kids in the primary school, the prevalence is 2.83/1000. Furthermore, in adolescents, it will rise to 3.5/1000 [1–3]. According to the latest date, approximately 50%-60% of congenital HL is caused by genetic factors [4, 5]. However, genetic disorder can also cause late-onset deafness in children and adolescents [2]. Both congenital or late-onset deafness caused by genetic disorder can be attributed to hereditary hearing loss. To date, more than 150 deafness-related genes and 6000 variants have been identified [6] (https://hereditaryhearingloss.org). The hearing loss genes and hot variants showed distinctly in different ethnic groups. Among these hearing loss genes, *GJB2* and *SLC26A4* gene variants were most common in Chinese deaf population [4]. For *GJB2* gene variants, the most frequent etiological factor of nonsyndromic hereditary hearing loss, the hotspots were described as c.235delC and c.109G>A, common in Asian, c.35delG, found in European, and c.71G > A, predominant in the Indian [7, 8]. Although *GJB2* and *SLC26A4* mutations account for a large part of causes of hereditary deafness, there are still a large number of known or unknown gene mutations that cause hearing loss. How to quickly and accurately detect causes of patients with suspected hereditary deafness is an important issue in the diagnosis and treatment of deafness.

Genetic testing is the gold standard for exploring the etiology of congenital and late-onset deafness. In addition, medical history and other auditory physiology tests are also very important. For hereditary deafness, genetic testing is a crucial step. Except for finding causes, it also could help diagnosis and intervention of syndromic hearing loss because some children only exhibited hearing loss without other symptoms at a young age. Besides, genetic testing was beneficial to protecting children with some variants such as m.1555A > G or variants in *KCNQ1* gene from avoidable risk factors [9–12]. DNA sequencing first described 44 years ago has evolved from single-mutation sequencing (Sanger sequencing) to high-throughput sequencing (Next generation sequencing, NGS) [5, 13]. Compared to Sanger sequencing, NGS was able to simultaneously sequence millions of small fragments at a reasonable cost and reduced runtime [13]. Targeted gene panel, a common NGS technique, has been widely applied in deaf population. It was targeted to the detection of variants in the massively known hearing loss genes. Considering the cost and time consuming, different panels were designed in clinical applications [1, 4, 12, 14, 15].

As the spectrum of gene mutations is distinct in different ethnic group, studying mutated character of Chinese deaf children and choosing the appropriate gene panel are critical for clinical diagnosis and treatment. In this study, we enrolled 137 Chinese children with sensorineural hearing loss to describe the molecular epidemiology by using 127 gene panel testing and 159 variants in 22 gene testing.

## 2. Materials and Methods

### 2.1. Patients and Samples

A total of 137 patients under the age of twelve with sensorineural hearing loss were recruited from the Wuhan Union Hospital from 2018 to 2020. All patients failed pass the neonatal hearing screening and underwent 127 gene panel testing (targeting the exon regions and exon-intron boundaries of 127 known deafness-causing nuclear genes as well as deafness-causing mitochondrial regions) or 159 variants in 22 gene testing (detecting a total of 159 hotspot variants in 22 known deafness-causing genes). Informed consent was obtained from all patients or their legal guardians. This study was approved by the Institutional Review Board of the BGI in accordance with the Declaration of Helsinki (1964). Written informed consent, clinical evaluations, and blood samples were obtained from all the participants or their legal guardians. The study of the protocols was approved by the review boards of the ethics committees of the Tongji Medical College of Huazhong University of Science and Technology.

Peripheral venous blood samples were collected from all recruited patients. Genomic DNA was obtained and purified using a QIAamp DSP DNA Blood Mini Kit (61104, Qiagen Inc., Valencia, CA, USA).

### 2.2. Library Preparation, Sequencing, and Bioinformatics

Fragmented DNA (a size of 350–400 base pairs) were prepared using Covaris LE220 ultrasonicator (Covaris Inc., Woburn, Massachusetts, USA). Then end-repair and adaptor ligation were performed for library construction. After array hybridization, elution, and postcapture amplification, targeted DNA fragments were sequenced on the BGISEQ-500 platform. After sequencing and quality control, the clean reads derived from targeted high-throughput sequencing were aligned to the GRCh37/hg19 using the BWA (Burrows Wheeler Aligner) Multi-Vision software package. After alignment, the variants of single-nucleotide variants (SNVs) and inserts and deletions (InDels) were detected by GATK software. Then, filtered SNVs and InDels were compared with NCBI GenBank database, 1000 Genomes, ESP6500, dbSNP, HGMD, and ExAC. Candidate variants were classified into pathogenic variants, likely pathogenic variants, variants of uncertain significance (VUS), likely benign variants, and benign variants according to the American College of Medical Genetics and Genomics–Association for Molecular Pathology (ACMG–AMP) guideline. The method described in this study partly reproduces the wording in our previous article [16–18].

## 3. Result

### 3.1. Study Samples

A total of 137 patients were included in this research, seventy-four patients were male and sixty-three were female. Sixty-three patients (46%) received genetic testing by 127 gene panel, while seventy-four patients (54%) received 159 variant testing (Table 1). Tested genes are provided in supplement (Table S1 and S2.)

### 3.2. Genetic Spectrum

Among the 63 received 127 gene panel test, ninety-two variants in 32 genes were detected, the top five genes were *GJB2* (33 patients), *SLC26A4* (16 patients), *MYO15A* (5 patients), *CDH23* (5 patients), and *OTOF* (5 patients) (Table 2).

Among the 74 who received 159 variant testing, twenty-one variants in 4 genes were detected; the patients with mutation of *GJB2*, *SLC26A4*, *GJB3*, and *MT-RNR1* were 45, 27, 2, and 1, respectively (Table 3).

### 3.3. Different Diagnosis between 127 Gene Panel Testing and 159 Variant Testing

**Figure 1:**
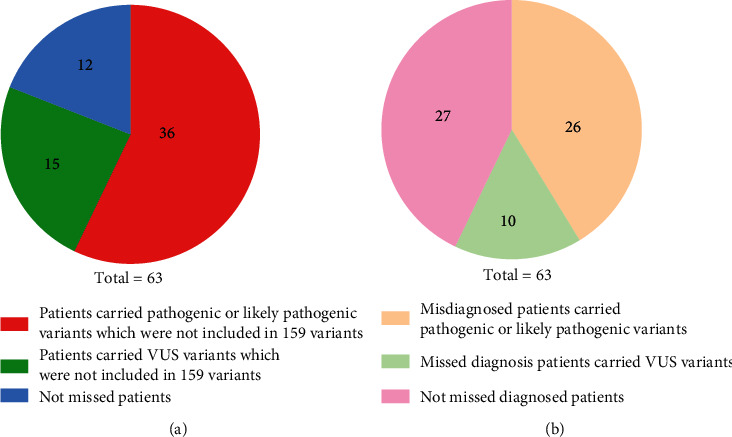
Analyze the genetic etiology of patients who received 127 gene panel testing compared with 159 variants. (a) 36 patients carried pathogenic or likely pathogenic variants which were not included in 159 variants, fifteen patients carried VUS variants which were not included in 159 variants, and 12 patients do not carry the variants which were not included in 159 variants. (b) 26 patients who carried pathogenic or likely pathogenic variants would misdiagnose if they were only detected by 159 variant testing, ten patients who carried VUS might miss diagnosis, and twenty-seven patients would not miss diagnosis.

Among the 63 patients who received 127 gene testing, fifty-one patients were found to carry variants which were not included in 159 variant testing, thirty-six of them had been found to carry pathogenic or likely pathogenic variants. If these patients received 159 variant testing only, in 36 of them, the genetic etiology of the hearing loss would be missed, and 26 of the patients were found to carry the pathogenic or likely pathogenic variants (Figure 1). Twenty-two deafness gene mutation which was not included in 159 variant testing was found in 25 patients; nine of them carried pathogenic or likely pathogenic variants of these 22 deafness genes. In these 22 deafness genes which were not included in 159 variant testing, eighty-four variants were found, ten of them were pathogenic or likely pathogenic (Table 4).

### 3.4. *GJB2* Variants

In 127 gene panel testing, six variants of *GJB2* were identified; c.109G>A, c.235delC, c.299_300del AT, c.176_191del GCTGCAAGAACGTGTG, c.139G>T, and c.88A>G were detected 18, 15, 5, 2, 1, and 1 times, respectively (Table 5). The patients who carried heterozygous variant of c.109G>A were the most, and secondary was heterozygous c.235delC mutation in 11 patients, and then was the homozygous c.109G>A mutation in 5 patients and homozygous c.235delC mutation in 4 patients. In the 11 patients who carried heterozygous c.235delC variants, four of them carried c.299_300del AT, 2 carried c.109G>A, and 2 carried c.176_191del GCTGCAAG AACGTGTG compound heterozygotes variants, respectively. In our study, a patient who carried a novel heterozygous variant was identified, the nucleotide change is c.88A>G, the protein change is p.Ile30Val, and the characterization of variant is VUS; this patient carried c.919-2A>G and c.1229C>T compound heterozygotes variants of SLC26A4 gene as well.

In 159 variant testing, eight variants of *GJB2* were identified; the frequency of these variants from high to low were c.235delC, c.299_300delAT, c.176_191del GCTGCAAGAACGTGTG, c.139G>T, c.187G>T, c.257C>G, c.427C>T, and c.428G>A; the check-out times of these variants were 36, 9, 5, 2, 1, 1, 1, and 1, respectively. Nineteen patients carried heterozygous c.235delC variants, five of them carried c.299_300delAT compound heterozygotes variants, three patients carried c.176_191del GCTGCAAGAACGTGTG compound heterozygotes variants, and one carried c.257C>G compound heterozygotes variants. The homozygous c.235delC patients were seventeen. c.299_300delAT heterozygous variant was identified in 9 patients, two of them carried c.176_191delGCTGCAAGAACGTGTG compound heterozygotes variants.

### 3.5. *SLC26A4* Variants

In 127 gene panel testing, seventeen variants were identified in *SLC26A4* gene, the most frequent variant detected was c.919-2A>G, eleven patients carried heterozygous c.919-2A>G variant, nine of them were compound heterozygotes variants with other SLC26A4 variants, and one homozygous c.919-2A>G was identified. Eight novel variants were identified in our research; c.1339delA, c.1519delT, c.164+1G>C, and c.2000T>C were likely pathogenic variants, while c.1340A>T, c.718C>T, c.765+5G>A, and c.208C>A were VUS variants (Table 6).

Twelve variants of *SLC26A4* were identified in 159 variant testing; the most common variant was c.919-2A>G same as the 127 gene panel testing, then was c.1229C>T and c.2168A>G. Nineteen patients carried c.919-2A>G variant, fourteen of them were heterozygous while 5 were homozygous.

## 4. Discussion

Hearing scanning combined with molecular genetic testing is an effective approach to diagnose hereditary hearing loss, while because of the high cost of genetic testing, such limited the usage of more comprehensive genetic testing. Through 127 gene panel testing, more genetic etiology of deaf children was identified. In 127 gene panel testing, thirty-two deafness genes were identified in patients. The five most frequent genes were *GJB2* (33 patients), *SLC26A4* (16 patients), *MYO15A* (5 patients), *OTOF* (5 patients), and *CDH23* (5 patients), while only four deafness genes were identified by 159 variant testing, and the frequency of these four genes was 45 (*GJB2*), 27 (*SLC26A4*), 2 (*GJB3*), and 1 (*MT-RNR1*), respectively. The genetic spectrum of 127 gene testing is consistent with the previous research in China [19]. The diagnostic rate of *GJB2* and *SLC26A4* is higher in 159 variant testing which may cause more mutant genes identified in 127 gene panel testing. Furthermore, eighty-four variants in 32 genes which were not included in 159 variant testing were identified, and ten variants of these were pathogenic or likely pathogenic. This result demonstrates that even though 159 variant testing contain hotspot deafness variants, it would still misdetect abundant of gene mutations.

Broader genetic testing can improve the diagnostic sensitivity and accuracy. By analyzing the patients who received 127 gene panel testing, we found that 25 patients had mutant genes which were not included in 159 variant testing, and nine of them carried pathogenic or likely pathogenic variants. Fifty-one patients carried variants that were not included in 159 variant testing, and thirty-six of them carried pathogenic or likely pathogenic variants. If these patients only received 159 variant testing, the genetic etiology would not be identified. This result revealed that even if the patients pass the traditional gene testing, they still could have genetic causes. Therefore, expanded gene testing should be done to identify the genetic etiology.

Moreover, accurate genetic testing can benefit patients in several aspects. Identifying genetic etiology can guide clinicians to determine treatment strategies. In our research, we found several patients carried variants in *CDH23*, *WFS1*, and *PCDH15* by 127 gene panel testing. As reported in previous research, some patients with above gene mutation may have a poor prognosis of cochlear implantation (CI) [20]. Besides, early genetic diagnostic of hearing loss would provide more information on nature, mode of inheritance, and implications of genetic disorders, which would help individuals and families make informed medical and personal decisions [14, 21]. Furthermore, the relationship between genotype and phenotype of hereditary hearing loss is complicated; finding novel variants would broaden the understanding of hereditary hearing loss.

Genetic testing should be optimized according to multiple dimensions. Firstly, the common detection of deafness genes and variants should vary by ethnicity and region. For example, in 127 gene panel testing, c.109G>A was the most frequent variant, then was the c.235delC. In our research, we did not select the ethnicity of the patients, which would influence the frequency of these variants. A previous research in Qinghai (China) has shown that c.109G>A was more common in minority patients, while c.235delC was more common in Han nationality [22]. Secondly, some gene mutation which might be associated with poor prognosis of CI such as *POU3F4*, *TMPRSS3*, *PJVK*, *CDH23*, and *PCDH15* should be added in genetic testing. The full sequence of these genes or at least the exon regions should be detected. If pathological mutations in these genes are missed, it may bring catastrophic consequences to patients and their families.

## 5. Conclusion

Congenital deafness is the number one congenital disease that endangers human health. The identification of the cause through hearing screening combined with genetic testing is an important basis for mechanism research, clinical intervention, and genetic counseling. With the development of sequencing technology, deeper and broader genetic testing by next-generation sequencing are efficient and affordable. More accurate and convenient genetic testing should be designed and recommended. It can increase the detection rate of patients with hereditary deafness, guide clinical treatment strategies, expand new deafness genes or variants, and provide help for future clinical work.

## Figures and Tables

**Table 1:** The basic information of patients recruited in this research.

	127 gene panel	159 variants	Total
Male	35	39	74
Female	28	35	63
Total	63	74	137

**Table 2:** The genetic spectrum of hereditary hearing loss patients detected by 127 gene panel testing.

Gene	Patients carried variant	Pathogenic variant	Likely pathogenic variant	VUS variant
*GJB2*	33	32	0	1
*SLC26A4*	16	15	0	1
*OTOF*	5	1	3	1
*MYO15A*	5	1	1	3
*CDH23*	5	0	2	3
*TECTA*	4	0	1	3
*MYO7A*	4	0	0	4
*WFS1*	3	1	0	2
*PTPRQ*	2	1	0	1
*COCH*	2	0	2	0
*GJB3*	2	0	1	1
*OTOA*	2	0	1	1
*USH2A*	2	0	1	1
*ESPN*	2	0	0	2
*TRIOBP*	2	0	0	2
*GRXCR1*	1	1	0	0
*ALMS1*	1	1	0	0
*COL4A3*	1	0	1	0
*ILDR1*	1	0	1	0
*ADGR V1*	1	0	0	1
*CLDN14*	1	0	0	1
*CRYM*	1	0	0	1
*DIAPH3*	1	0	0	1
*EDNRB*	1	0	0	1
*LOXHD1*	1	0	0	1
*MITF*	1	0	0	1
*MYH14*	1	0	0	1
*PCDH15*	1	0	0	1
*PDSS1*	1	0	0	1
*SLC17A8*	1	0	0	1
*TJP2*	1	0	0	1
*TMC1*	1	0	0	1

**Table 3:** The genetic spectrum of hereditary hearing loss patients detected by 159 variant testing.

Gene	Patients with mutant gene	Percentage
*GJB2*	45	0.608108
*SLC26A4*	27	0.364865
*GJB3*	2	0.027027
*MT-RNR1*	1	0.013514

**Table 4:** The variants would be missed if tested by 159 variant testing.

Gene	Variant	Effect on protein	Characterization of variant
*ADGR V1*	c.11704A>G	p.Met3902Val	VUS
	c.2899-10T>A		VUS
*ALMS1*	c.2035C>T	p.Arg679Ter	Pathogenic
*CDH23*	c.5067+1G>A		Likely pathogenic
	c.6604G>A	p.Asp2202Asn	Likely pathogenic
	c.1765G>A	p.Asp589Asn	VUS
	c.2368A>G	p.Met790Val	VUS
	c.3262G>A	p.Val1088Met	VUS
	c.4859T>A	p.Val1620Glu	VUS
	c.5051G>A	p.Arg1684His	VUS
	c.805C>T	p.Arg269Trp	VUS
*CLDN14*	c.449C>T	p.Pro150Leu	VUS
	c.694G>A	p.Gly232Arg	VUS
*COCH*	c.812_813insG	p.Val271Valfs X5	Likely pathogenic
COL4A3	c.4755+1G>A		Likely pathogenic
*CRYM*	c.849C>T	p.His283His	VUS
*DIAPH3*	c.3543_3544i nsC	p.Pro1181 Profs∗4	VUS
*EDNRB*	c.758G>A	p.Arg253Gln	VUS
*ESPN*	c.1828G>C	p.Ala610Pro	VUS
	c.2069C>T	p.Ser690Leu	VUS
*GJB2*	c.109G>A	p.Val37Ile	Pathogenic
	c.139G>T	p.Glu47Ter	Pathogenic
	c.88A>G	p.Ile30Val	VUS
*GJB3*	c.580G>A	p.Ala194Thr	Likely pathogenic
	c.595A>G	p.Ile199Val	VUS
*GRXCR1*	c.784C>T	p.Arg262Ter	Pathogenic
*ILDR1*	c.206C>A	p.Pro69His	Likely pathogenic
*LOXHD1*	EX31 DUP		VUS
*MITF*	c.950G>C	p.Arg317Thr	VUS
*MYH14*	c.4641C>T	p.Asp1547Asp	VUS
*MYO15A*	c.10251_10253 delCTT		Pathogenic
	c.4642G>A	p.Ala1548Thr	Pathogenic
	c.8702_8703i nsT	p.Pro2901Pro fsX25	Likely pathogenic
	c.3239G>A	p.Arg1080His	VUS
	c.3743G>A	p.Arg1248Gln	VUS
	c.5681T>C	p.Leu1894Pro	VUS
	c.6340G>A	p.Val2114Met	VUS
*MYO7A*	c.1901G>A	p.Arg634Gln	VUS
	c.1945C>T	p.Arg649Trp	VUS
	c.6235C>T	p.Arg2079Trp	VUS
	c.6470T>C	p.Ile2157Thr	VUS
*OTOA*	c.943delT	p.Ser315Profs ∗5	Likely pathogenic
	c.1172C>T	p.Ser391Leu	VUS
	c.2359G>T	p.Glu787Ter	VUS
*OTOF*	c.5816G>A	p.Arg1939Gln	Pathogenic
	c.4023+1G>A		Likely pathogenic
	c.5098G>C	p.Glu1700Gln	Likely pathogenic
	c.1194T>A	p.Asp398Glu	VUS
	c.3429G>A	p.Arg1143Arg	VUS
*PCDH15*	c.2563C>A	p.Arg855Arg	VUS
*PDSS1*	EX1 DUP		VUS
*PTPRQ*	c.6475C>T	p.Arg2159Ter	Pathogenic
	c.6025-4_6025-2delACA		VUS
	c.6293T>C	p.Leu2098Ser	VUS
*SLC17A8*	c.1117T>C	p.Leu373Leu	VUS
	c.243G>A	p.Met81Ile	VUS
*SLC26A4*	c.1079C>T	p.Ala360Val	Pathogenic
	c.1339delA	p.Lys447Serfs∗8	Pathogenic
	c.1519delT	p.Leu507∗	Pathogenic
	c.164+1G>C		Pathogenic
	c.2000T>C	p.Phe667Ser	Pathogenic
	c.1001+5G>T		VUS
	c.1087A>C	p.Ile363Leu	VUS
	c.1340A>T	p.Lys447Met	VUS
	c.208C>A	p.Pro70Thr	VUS
	c.678T>C	p.Ala226Ala	VUS
	c.718C>T	p.Leu240Phe	VUS
	c.765+5G>A		VUS
*TECTA*	c.640G>T	p.Gly214Ter	Likely pathogenic
	c.235G>C	p.Val79Leu	VUS
	c.2936T>C	p.Phe979Ser	VUS
	c.576G>A	p.Thr192Thr	VUS
*TJP2*	c.474G>T	p.Arg158Ser	VUS
*TMC1*	c.1810C>G	p.Arg604Gly	VUS
	c.589G>A	p.Gly197Arg	VUS
*TRIOBP*	c.2133T>C	p.Pro711Pro	VUS
	c.2314T>C	p.Cys772Arg	VUS
	c.-60-1G>C		VUS
*USH2A*	c.6937G>T	p.Gly2313Cys	Likely pathogenic
	c.10740+7G>A		VUS
	c.7230A>T	p.Val2410Val	VUS
*WFS1*	c.2051C>T	p.Ala684Val	Pathogenic
	c.125G>T	p.Arg42Leu	VUS
	c.2458G>A	p.Gly820Ser	VUS

**Table 5:** Variants frequency in *GJB2* gene.

Variants	Homozygous	Heterozygous	Total
*Testing by 127 gene panel*			
c.109G>A	5	13	18
c.235delC	4	11	15
c.299_300del AT	1	4	5
c.176_191del GCTGCAAG AACGTGTG	0	2	2
c.139G>T	1	0	1
c.88A>G	0	1	1
*Testing by 159 variant testing*			
c.235delC	17	19	36
c.299_300delAT	0	9	9
c.176_191delGCTGCAAGAACGTGTG	0	5	5
c.139G>T	2	0	2
c.187G>T	0	1	1
c.427C>T	1	0	1
c.428G>A	0	1	1
c.257C>G	0	1	1

**Table 6:** Variants frequency in *SLC26A4* gene.

Variants	Homozygous	Heterozygous	Total
*Testing by 127 gene panel*			
c.919-2A>G	1	11	12
c.2000T>C	0	2	2
c.1087A>C	0	1	1
c.1174A>T	0	1	1
c.1339delA	0	1	1
c.164+1G>C	0	1	1
c.718C>T	0	1	1
c.1226G>A	0	1	1
c.678T>C	0	1	1
c.1519delT	0	1	1
c.1229C>T	0	1	1
c.1340A>T	0	1	1
*Testing by 159 variant testing*			
c.919-2A>G	5	14	19
c.1229C>T	0	5	5
c.2168A>G	0	4	4
c.1174A>T	0	1	1
c.1226G>A	0	1	1
c.1336C>T	0	1	1
c.1343C>A	0	1	1
c.1343C>T	0	1	1
c.1594A>C	0	1	1
c.1692dupA	0	1	1
c.1707+5G >A	0	1	1
c.589G>A	0	1	1

## Data Availability

The data which support the conclusions of our study is upon the request.

## Supplementary Materials

Supplementary Materials See Supplementary Tables S1 and S2 in the Supplementary Material. Tested genes among 127 gene panel testing are provided in Table S1, and tested variants in 159 variant testing are provided in Table S2.
