# Supplementary material for: Hearing Screening Combined with Target Gene Panel Testing Increased Etiological Diagnostic Yield in Deaf Children
Source: Neural Plast. 2021 Jul 23;2021:6151973. doi: 10.1155/2021/6151973 (PMC8324351; doi:10.1155/2021/6151973)
Supplement: Supplementary Materials — See Supplementary Tables S1 and S2 in the Supplementary Material. Tested genes among 127 gene panel testing are provided in Table S1, and tested variants in 159 variant testing are provided in Table S2. [file 6151973.f1.docx]

**Table S1. 127 gene panel testing**

| Type of hearing loss | Gene |
| --- | --- |
| Autosomal recessive non-syndromic hearing loss | *GJB2, GJB6, MYO7A, MYO15A, FOXI1, KCNJ10, SLC26A4, TMIE, TMC1, TMPRSS3, OTOF, CDH23, ATP2B2, GIPC3, STRC, OTOG, USH1C, TECTA, OTOA, PCDH15, RDX, GRXCR1, TRIOBP, CLDN14, MYO3A, DFNB31, ESRRB, ESPN, MYO6, GJA1, HGF, ILDR1, MARVELD2, DFNB59, SLC26A5, LRTOMT, LHFPL5, BSND, MSRB3, LOXHD1, TPRN, GPSM2, PTPRQ, SERPINB6, GJB3* |
| X-link hereditary hearing impairment | *PRPS1, POU3F4, SMPX* |
| Syndromic hearing impairment | *SERAC1, PDSS1, FGFR3, FGFR1, FGFR2, PHEX, DLX5, TNFRSF11B, COL2A1, COL11A1, COL9A1, COL9A2, COL4A3, COL4A4, COL4A5, BSND, SOX9, PAX2, GATA3, SLC19A2, IGF1,*  *PAX3, MITF, SNAI2, EDNRB, EDN3, SOX10, HOXA1, SOBP, EYA1, SIX5, SIX1, CHD7, SEMA3E, SMAD4, FGF3, TCOF1, PRRX1, GLI3, HOXA2, KCNQ1, KCNE1, CACNA1D, ALMS1, LRP2, TIMM8A, NDP, WFS1, OPA1, SLC4A11, MYO7A, USH1C, CDH23, PCDH15, USH1G, USH2A, GPR98, PDZD7, DFNB31, CLRN1, MT-TK, MT-TE, MT-TL1, SLC26A4, KCNJ10, FOXI1* |
| Autosomal dominant non-syndromic hearing impairment | *ACTG1, CCDC50, CEACAM16, COCH, CRYM, DFNA5, DIABLO, DIAPH1, DSPP, EYA4, GJB2, GJB3, GJB6, GRHL2, KCNQ4, MIR96, MYH14, MYH9, MYO1A, MYO6, MYO7A, POU4F3, SIX1, SLC17A8, TECTA, TJP2, TMC1, WFS1, DIAPH3* |
| Maternally inherited hearing impairment | *MT-RNR1, MT-TS1* |

**Table S2. 159 variants in 22 genes testing**

| Number | Gene | Mutation | Number | Gene | Mutation |
| --- | --- | --- | --- | --- | --- |
| 1 | *CDH23* | c.902G>A | 38 | *GJB2* | c.269T>C |
| 2 | *COL11A1* | c.4171-2A>G | 39 | *GJB2* | c.283G>A |
| 3 | *DFNA5* | c.1183+4A>G | 40 | *GJB2* | c.298C>T |
| 4 | *DFNB59* | c.547C>T | 41 | *GJB2* | c.34G>C |
| 5 | *DSPP* | c.52G>T | 42 | *GJB2* | c.35G>A |
| 6 | *GJB2* | c.155_158delTCTG | 43 | *GJB2* | c.35G>T |
| 7 | *GJB2* | c.176_191delGCTG CAAGAACGTGTG | 44 | *GJB2* | c.365A>T |
| 8 | *GJB2* | c.235delC | 45 | *GJB2* | c.427C>T |
| 9 | *GJB2* | c.269dupT | 46 | *GJB2* | c.428G>A |
| 10 | *GJB2* | c.280_284dupCACGT | 47 | *GJB2* | c.439G>A |
| 11 | *GJB2* | c.290dupA | 48 | *GJB2* | c.44A>C |
| 12 | *GJB2* | c.299_300delAT | 49 | *GJB2* | c.487A>C |
| 13 | *GJB2* | c.310_323delAG GAAGTTCATCAA | 50 | *GJB2* | c.487A>G |
| 14 | *GJB2* | c.535G>A | 51 | *GJB2* | c.506G>A |
| 15 | *GJB2* | c.35delG | 52 | *GJB2* | c.50C>T |
| 16 | *GJB2* | c.35dupG | 53 | *GJB2* | c.313_326delAAGTTCATCAAGGG |
| 17 | *GJB2* | c.504_505insAAGG | 54 | *GJB2* | c.551G>A |
| 18 | *GJB2* | c.508_511dupAACG | 55 | *GJB2* | c.551G>C |
| 19 | *GJB2* | c.509dupA | 56 | *GJB2* | c.596C>T |
| 20 | *GJB2* | c.512_513insAACG | 57 | *GJB2* | c.605G>T |
| 21 | *GJB2* | c.564_565delGA | 58 | *GJB2* | c.94C>T |
| 22 | *GJB2* | c.575_576delCA | 59 | *GJB2* | c.95G>A |
| 23 | *GJB2* | c.576delA | 60 | *GJB2* | c.95G>T |
| 24 | *GJB2* | c.632_633delGT | 61 | *GJB2* | c.132G>A |
| 25 | *GJB2* | c.99delT | 62 | *GJB2* | c.139G>T |
| 26 | *GJB2* | c.104T>G | 63 | *GJB2* | c.169C>T |
| 27 | *GJB2* | c.107T>C | 64 | *GJB2* | c.230G>A |
| 28 | *GJB2* | c.132G>C | 65 | *GJB2* | c.231G>A |
| 29 | *GJB2* | c.134G>A | 66 | *GJB2* | c.238C>T |
| 30 | *GJB2* | c.164C>A | 67 | *GJB2* | c.370C>T |
| 31 | *GJB2* | c.175G>A | 68 | *GJB2* | c.598G>T |
| 32 | *GJB2* | c.187G>T | 69 | *GJB2* | c.71G>A |
| 33 | *GJB2* | c.224G>A | 70 | *GJB3* | c.520G>A |
| 34 | *GJB2* | c.229T>C | 71 | *GJB3* | c.547G>A |
| 35 | *GJB2* | c.250G>A | 72 | *GJB3* | c.538C>T |
| 36 | *GJB2* | c.250G>C | 73 | *KCNJ10* | c.491C>T |
| 37 | *GJB2* | c.257C>G | 74 | *MT-RNR1* | m.1494C>T |
| 75 | *MT-RNR1* | m.1555A>G | 118 | *SLC26A4* | c.1997C>T |
| 76 | *MT-RNR1* | m.961T>C | 119 | *SLC26A4* | c.2015G>A |
| 77 | *MT-TL1* | m.3243A>G | 120 | *SLC26A4* | c.2027T>A |
| 78 | *MT-TS1* | m.7445A>G | 121 | *SLC26A4* | c.2162C>T |
| 79 | *MYO15A* | c.8183G>A | 122 | *SLC26A4* | c.2168A>G |
| 80 | *MYO15A* | c.8767C>T | 123 | *SLC26A4* | c.230A>T |
| 81 | *MYO7A* | c.731G>C | 124 | *SLC26A4* | c.259G>T |
| 82 | *MYO7A* | c.1996C>T | 125 | *SLC26A4* | c.367C>T |
| 83 | *MYO7A* | c.2005C>T | 126 | *SLC26A4* | c.439A>G |
| 84 | *MYO7A* | c.700C>T | 127 | *SLC26A4* | c.589G>A |
| 85 | *MYO7A* | c.133-2A>G | 128 | *SLC26A4* | c.626G>T |
| 86 | *OTOF* | c.3624delG | 129 | *SLC26A4* | c.679G>C |
| 87 | *PCDH15* | c.1088delT | 130 | *SLC26A4* | c.707T>C |
| 88 | *PCDH15* | c.1036G>T | 131 | *SLC26A4* | c.716T>A |
| 89 | *SLC26A4* | c.1181_1183delTCT | 132 | *SLC26A4* | c.754T>C |
| 90 | *SLC26A4* | c.1198delT | 133 | *SLC26A4* | c.920C>T |
| 91 | *SLC26A4* | c.1238delA | 134 | *SLC26A4* | c.1336C>T |
| 92 | *SLC26A4* | c.1341delG | 135 | *SLC26A4* | c.1343C>A |
| 93 | *SLC26A4* | c.1520delT | 136 | *SLC26A4* | c.1540C>T |
| 94 | *SLC26A4* | c.1547dupC | 137 | *SLC26A4* | c.1554G>A |
| 95 | *SLC26A4* | c.1555_1556delAA | 138 | *SLC26A4* | c.170C>A |
| 96 | *SLC26A4* | c.1586delT | 139 | *SLC26A4* | c.170C>G |
| 97 | *SLC26A4* | c.1692dupA | 140 | *SLC26A4* | c.1768A>T |
| 98 | *SLC26A4* | c.1746delG | 141 | *SLC26A4* | c.249G>A |
| 99 | *SLC26A4* | c.349delC | 142 | *SLC26A4* | c.1615-1G>A |
| 100 | *SLC26A4* | c.365dupT | 143 | *SLC26A4* | c.1615-2A>G |
| 101 | *SLC26A4* | c.387delC | 144 | *SLC26A4* | c.165-1G>A |
| 102 | *SLC26A4* | c.1264-12T>A | 145 | *SLC26A4* | c.919-2A>G |
| 103 | *SLC26A4* | c.1707+5G>A | 146 | *SLC26A4* | c.1341+1G>C |
| 104 | *SLC26A4* | c.1804-6G>A | 147 | *SLC26A4* | c.2089+1G>A |
| 105 | *SLC26A4* | c.1151A>G | 148 | *SLC26A4* | c.600+2T>A |
| 106 | *SLC26A4* | c.1160C>T | 149 | *SOX10* | c.565G>T |
| 107 | *SLC26A4* | c.1173C>A | 150 | *SOX10* | c.621C>G |
| 108 | *SLC26A4* | c.1174A>T | 151 | *TCOF1* | c.386_387delCA |
| 109 | *SLC26A4* | c.1226G>A | 152 | *TCOF1* | c.422dupA |
| 110 | *SLC26A4* | c.1229C>T | 153 | *TCOF1* | c.497_500delATAC |
| 111 | *SLC26A4* | c.1334T>G | 154 | *TMC1* | c.100C>T |
| 112 | *SLC26A4* | c.1343C>T | 155 | *USH1G* | c.84dupC |
| 113 | *SLC26A4* | c.1540C>A | 156 | *USH1G* | c.113G>A |
| 114 | *SLC26A4* | c.1541A>G | 157 | *WFS1* | c.1511C>T |
| 115 | *SLC26A4* | c.1586T>G | 158 | *WFS1* | c.1433G>A |
| 116 | *SLC26A4* | c.1594A>C | 159 | *WHRN* | c.1267C>T |
| 117 | *SLC26A4* | c.1975G>C |  |  |  |
